# Factors Associated With Health Care Utilization Among Working Caregivers

**DOI:** 10.1093/geroni/igaa057.2551

**Published:** 2020-12-16

**Authors:** Tiffany Washington, Eunhye Kim, George Mois, Matthew Smith

**Affiliations:** 1 University of Georgia, Athens, Georgia, United States; 2 Augusta University, Augusta, Georgia, United States; 3 University of Georgia, Lawrenceville, Georgia, United States; 4 Texas A&M University, College Station, Texas, United States

## Abstract

This study examined factors associated with health care utilization among working family caregivers using data from the 2013 Regional Healthcare Partnership – Region 17 Health Assessment survey. Anderson’s Behavioral Model of Health Services Use guided the selection of variables. Chi-square or t-test were computed to compare statistically significant differences between caregivers who did and did not utilize health care, and logistic regression was employed to identify factors associated with health care utilization. Of the 220 working caregivers, 41.8% put off their health care primarily because they could not miss work. Age, days pain limited activity, and days not having enough rest or sleep were associated with health care utilization. Paid family leave could mitigate the challenge of simultaneously managing employment responsibilities, caregiving tasks, and health needs of working family caregivers.

